# A map-based logistic neuron model: an efficient way to obtain many different neural behaviors

**DOI:** 10.1186/1471-2202-15-S1-P24

**Published:** 2014-07-21

**Authors:** Rafael V Stenzinger, Jheniffer J Gonsalves, Mauricio Girardi-Schappo, Marcelo HR Tragtenberg

**Affiliations:** 1 Physics Department, Federal University of Santa Catarina, Florianópolis, SC, 88040-900, Brazil

## 

The KTz neuron model [1-5] is a map with three dynamic variables: the neuron membrane potential ($x_{t}$), a recovery variable ($y_{t}$) and a slow adaptive current ($z_{t}$), given by the following equations:

$$x_{t+1}=\text{tanh}\left(\frac{x_{t}-Ky_{t}+z_{t}+I_{t}}{T}\right),$$

$$y_{t+1}=x_{t},$$

$$z_{t+1}=\left(1-\delta \right)z_{t}-\lambda \left(x_{t}-x_{R}\right),$$

where  δ is related to the refractory period, $x_{R}$ is the reversal potential,  λ controls the burst sizes, $I_{t}$ is an external current and  K and  T are gain parameters for neuron self-interactions. The hyperbolic tangent is biologically plausible, since it is a sigmoidal function that saturates at large absolute inputs.

This model exhibits a rich repertoire of dynamical behaviors, especially those of excitable neurons: bursting, fast and regular spiking, spikes with plateau, type I and II excitability, chaotic firing, among others [1-4]. When associated in networks, they exhibit synchronization, power law avalanches, criticality, etc. [4,5].

However, hyperbolic tangent is a computationally expensive sigmoidal function. Considering the need of a good trade-off between computational efficiency and biological relevance [6-8], we then propose the use of the logistic function $\text{f}\left(u\right)=u/\left(1+\left|u\right|\right)$, which displays a similar asymptotic behavior and is computationally faster. We call it the KTz logistic model. A similar model has been recently used to study the unlearning hypothesis in REM sleep [9].

We determine the complete phase diagram of the KTz logistic model with details for $z_{t}=\delta =\lambda =0$. This phase diagram is similar to the hyperbolic tangent KTz under the same conditions [5], although it is more analytically tractable. We also determine the phase diagram of the logistic KTz model with δ=λ=0 and $z_{t}\neq 0$ and compare with the hyperbolic tangent KTz [1,4].

The myriad of dynamical behaviors of the logistic KTz model (δ≠0 and λ≠0) and its  T versus $x_{R}$ phase diagram are also described and compared with those of the hyperbolic tangent KTz [1-4], showing the regions of cardiac like behavior, bursting, fast and slow spiking and fixed point. A detailed analysis of the order of the transitions between these regimes is also made.

The preliminary results of networks of logistic KTz models connected by map chemical synapses [2,4,5] are displayed and compared with those of hyperbolic KTz model. Finally, we make qualitative and quantitative comparisons between computational efficiency of the hyperbolic tangent and the logistic KTz models, for many different behaviors of the studied neurons.
